# Drinking Expectancies among Chinese Young Adults: A Qualitative Study from Hong Kong

**DOI:** 10.3390/ijerph191911865

**Published:** 2022-09-20

**Authors:** Rufina H. W. Chan, Dong Dong, Jean H. Kim

**Affiliations:** 1 The Jockey Club School of Public Health and Primary Care, The Chinese University of Hong Kong, Prince of Wales Hospital, Shatin, New Territories, Hong Kong SAR, China; 2 Shenzhen Research Institute, The Chinese University of Hong Kong, Shenzhen 518000, China

**Keywords:** alcohol, marketing, qualitative research, focus groups, China

## Abstract

Background: Alcohol expectancies, i.e., the perceived consequences of drinking, have been reported to be important factor in predicting drinking behaviors. However, studies in the Asia region were largely limited to school-based samples. This study aimed to be the first to explore drinking expectancies among urban Chinese young adults. Methods: In 2020, eight focus group discussions were conducted with Hong Kong Chinese young adults aged 18–34 (*n* = 53). The participants included heavy drinkers, light drinkers, and non-drinkers from a wide range of occupations and educational backgrounds. Thematic analysis was conducted to uncover common alcohol expectancies. Results: Six themes emerged from this study. Four themes that were commonly reported in the literature were the negative consequences of drinking, social bonding, confidence enhancement, and tension reduction. The study also uncovered two culturally relevant alcohol expectancies: health benefits and business drinking expectancies. In contrast to Western samples, Chinese young adults did not report drinking expectancies related to cognitive enhancement or increased sexual interest. Conclusion: Alcohol harm reduction strategies will need to address the positive drinking expectancies uncovered in this study. Future policy discussions in this emerging alcohol market region should consider greater scrutiny of the role of alcohol marketing in the propagation of positive drinking expectancies.

## 1. Introduction

According to the World Health Organization (WHO), approximately 3 million deaths per year and 5.1% of disability-adjusted life years worldwide are attributed to the harmful use of alcohol [1]. In the past two decades, drinking levels in traditionally low-consumption regions, such as WHO-South East Asia and WHO-Western Pacific regions, have been steadily rising [1]. By 2025, these regions are projected to have the highest increase in per capita drinking levels and rising levels of alcohol-related harms [1]. Currently, more than 15% of the total deaths among Chinese young men and 9% of deaths among women aged 15–29 years were attributable to alcohol use [2]. These data indicate the need for a better understanding of the factors that influence drinking among young people in regions with a continuing rise in per capita drinking levels. Due to the large cultural differences in drinking habits, studies conducted in countries such as the United States cannot be directly applied to regions such as China.

Alcohol expectancies, i.e., the perceived positive or negative consequences of consuming alcohol, were widely used to explain drinking behaviors, particularly among younger-aged drinkers. The expectancy theory posits that expectancies are formed in a memory-based cognitive learning process whereby a specific behavior becomes associated with certain consequences [3]. Unsurprisingly, alcohol expectancies were noted to be highly influenced by peers and mass media [4,5,6,7,8,9]. The majority of past alcohol expectancies studies were, however, conducted on Western samples using instruments that were originally validated for Western populations, such as the Alcohol Expectancies Questionnaire (AEQ) and Drinking Expectancies Questionnaire (DEQ) [10,11,12]. These studies consistently noted that young people who hold positive alcohol expectancies had higher drinking levels, increased likelihood of risky drinking behaviors, and greater intention to drink in the future [13,14,15,16,17,18,19,20]. Drinking expectancy research in East Asian populations primarily focused on school-aged adolescent samples [21,22,23,24,25,26]. A study of Mongolian Chinese secondary school students noted expectancies that were not present in Western samples (e.g., drinking as a social courtesy) [22]. A cross-cultural study also noted that Thai adolescents possessed various drinking expectancies based on their Buddhist religion [23].

Although there is no consensus definition of young adulthood, this life stage can range from the legal age of majority through one’s twenties and even one’s thirties [27,28]. In countries for which there is a statutory drinking age, the overwhelming majority allow alcohol purchases by those 18 years of age and older [29]. Among young adults in China, there has been a rapid uptake of drinking and a notably high prevalence of risky drinking behaviors [30,31,32]. However, studies in East Asia that explored drinking expectancies in young adults were mainly limited to university-based samples [33,34,35,36,37]. The authors were able to identify only two non-school-based studies of drinking motives among Chinese young adults. A cross-country (Nigeria, China, Uruguay, and Russia) survey of heavy episodic drinkers between 18–34 years of age found that the motivations for drinking differ greatly in each location [38]. It was noted that drinkers from Wuhan, China, were more likely to associate heavy drinking with negative consequences, such as aggression, as compared with the other regions. Another study from China of drinkers between 18–34 years of age noted that rather than personal/psychological motives, younger adult drinkers attached greater importance to social/celebratory reasons for drinking as a way to show friendship [39]. The authors of the study concluded that research based on Western models of drinking motivations may not fully capture the reasons for drinking in Chinese populations and called for more detailed studies of Chinese populations.

Hong Kong is a high-income Chinese city of about 7.5 million people and a major financial hub of Asia [40]. Since 2008, all beer/wine import taxes have been abolished, which was soon followed by widespread alcohol promotion and increased population drinking levels [41,42]. The most recent data indicate that the 25–34 age group had the highest proportion of drinkers and binge drinkers and a high risk of alcohol-related harms [43,44,45,46,47], and is, therefore, a target group for alcohol harm reduction strategies. The current literature lacks studies that examined drinking expectancies among young adults, who have a higher prevalence of drinking and binge drinking than any other age group in the East Asian region. Young adults represent a vulnerable group as the brain is still not completely developed [48]. Moreover, drinking in this age group has been associated with the use of psychotropic drugs and risky behaviors, such as drunk driving [49,50,51]. The limited research on alcohol expectancies focused on school-aged samples without taking account of the changes in the drinking environment and contexts after one enters the workforce. The authors hypothesized that there will be cultural-specific drinking expectancies that emerge in young adults in the region that are not currently examined by drinking expectancy instruments developed in Western countries. To address the research gap and to fully capture drinking-related beliefs that may not be captured in currently used instruments, this study aimed to explore the drinking-related expectancies among Chinese young adults in Hong Kong. This study was the first to examine drinking expectancies in this population using qualitative methods, which allowed the respondents to express their views without preconceived response choices. The study results will help to inform regional strategies for reducing harmful drinking.

## 2. Materials and Methods

In order to explore drinking expectancies in Chinese young adults, we conducted eight focus group discussions during October and November 2020 (*n* = 53) in Hong Kong. Focus groups encourage interaction between participants and are especially useful for exploring topics that are influenced in part by social norms and expectations, such as alcohol consumption. Due to social distancing restrictions of the COVID-19 pandemic, all focus groups were conducted online and recorded with the permission of the participants for later transcription and data analysis.

### 2.1. Recruitment and Implementation

**Figure 1:**
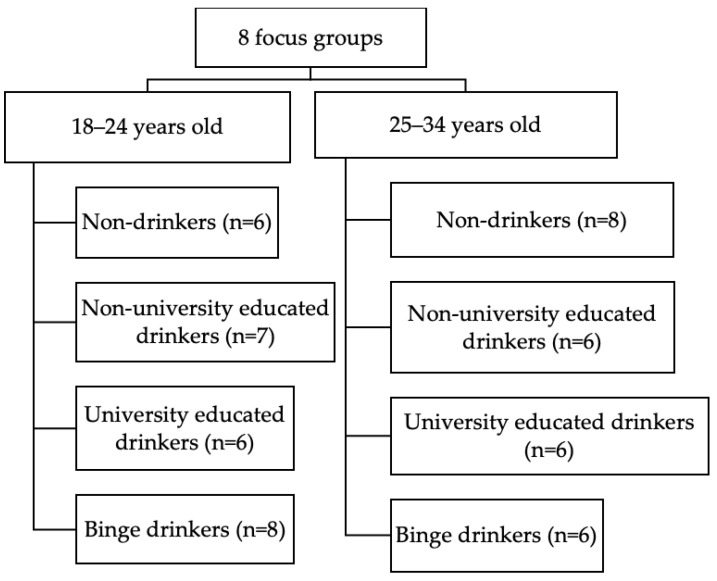
Number of focus groups conducted by age group and drinking category (*n* = 53).

Participant recruitment was conducted via multiple channels, including advertisements shared via various social media platforms, such as Facebook, Instagram, and LinkedIn; university email lists; and word-of-mouth. Young Hong Kong Chinese adults were initially sampled using maximum variability sampling to reflect a wide spectrum of occupations and drinking patterns across the 18–34 age group. In maximum variation sampling, a wide range of individuals is purposively selected such that all or most types of individuals are selected for the inquiry, allowing for multiple perspectives of individuals to be presented [52]. We conducted focus groups in the 18–24 and 25–34 age strata since young adults in their late twenties and early thirties would be likely to have more years of work experience, may be more likely to be married, and have more drinking-related experiences than those who were more likely to be still in school. In each of these 2 age strata, we included 1 focus group with each of the following drinking categories: (1) nondrinkers (individuals who had not drunk in the past year or had never consumed a full serving of alcohol), (2) non-university-educated drinkers (who did not binge drink), (3) university-educated drinkers (who did not binge drink), and (4) past-month binge drinkers (drinkers consuming 5+ servings for men or 4+ servings for women in one occasion) or habitual weekly drinkers (see Figure 1). The non-binge/non-weekly drinking category was stratified by educational attainment since education level has been associated with different drinking patterns/contexts [53,54].

The topic guide was pilot tested on 4 individuals from each drinking category and revised based on the feedback of the respondents.

Each focus group comprised 6–8 participants. The discussion lasted approximately 90 min and was led by a facilitator and notetaker. The moderator facilitated the discussions during the focus group, while the notetaker tracked the non-verbal cues and took notes. After each focus group discussion, both the facilitator and notetaker met and drafted a summary of the discussion to aid in later data analysis. The respondents were also asked to complete a demographic information form at the start of the study. Due to the social distancing restrictions of the COVID-19 pandemic, all focus groups were conducted online and recorded on the Zoom platform with informed consent [55]. Each participant received an HKD 200 (approximately USD 25) cash voucher for their participation.

### 2.2. Topic Guide

Participants were first informed of the interviewer’s aim of exploring drinking-related perceptions and beliefs about the consequences of alcohol consumption. The topic guide, which was designed based on a model employed by the RAND Institute to facilitate the thought process, began with broad questions, and then gradually spiraled into more specific questions related to the study objectives [56]. The topic guide included questions about attitudes and perspectives around drinking, personal histories of alcohol use, and alcohol-related expectancies. After the respondents discussed their own expectancies, they were then asked to examine a Chinese-translated Drinking Expectancies Questionnaire (DEQ), which is a commonly used instrument for assessing drinking expectancies and is comprised of five domains (negative consequences, increased confidence, sexual enhancement, cognitive enhancement, and tension reduction) [11]. The DEQ had previously undergone a translation/English back-translation process by bilingual researchers. Participants were asked to discuss whether they agreed with the items in each subscale and whether they find such expectancies to be relevant to the drinking behavior of themselves and their peer group. Each respondent was also asked to comment on ten local alcohol advertisements published by well-known alcohol brands and local drinking venues, which were used as visual aids for the focus group. The focus groups did not incorporate a formal educational component, but facilitators mentioned recent evidence contradicting the purported health benefits of alcohol to participants. The participant sample included drinkers and non-drinkers, but many non-drinkers discussed their views based on past alcohol consumption and experiences relating to peers who drink.

### 2.3. Data Analysis

All focus group discussions were recorded via Zoom; audio recordings were transcribed verbatim by the research team. All transcribed data were anonymized and imported into NVivo version 9 [57] for thematic analysis. We coded the material in all transcripts related to our research questions, identifying patterns across and between focus groups and interview transcripts.

The data analysis was conducted using a combination of inductive and deductive approaches. A pair of researchers read the transcripts repeatedly and independently and noted any potential important key quotes when categorizing the alcohol expectancy. The pair then compared notations for each transcript and resolved differences through discussion until they reached a consensus coding for each group’s transcript. All the alcohol-generated expectancies were first examined deductively and compared with the DEQ instrument [11]. Expectancies that aligned with the current DEQ subscales were retained. The verbatim transcripts were then analyzed using the inductive coding approach. Additional expectancies that emerged were then categorized and conceptualized into broad themes. The codes for within age group and within drinking category were also compared and discussed to examine whether there were similarities or differences in expectancies between the groups. Transcripts were coded in Chinese by bilingual coders and key quotes reported in this study were translated into English.

## 3. Results

### 3.1. Focus Group Participant Demographics

A total of 53 young adults participated in eight online focus group discussions during October and November 2020 and their characteristics are listed in Table 1.

### 3.2. Culturally Relevant Drinking-Related Expectancies

During the focus group discussion of drinking expectancies, which included viewing some recent advertisements and a review of a commonly used drinking expectancy instrument, one negative domain and five positive domains of drinking-related expectancies emerged.

#### 3.2.1. Expectancy 1: Short-Term Negative Consequences

All the participants perceived some level of negative impact of alcohol consumption. This drinking expectancy showed a high degree of consensus. The negative consequences mentioned were mostly limited to short-term adverse effects. Physical effects (such as the loss of consciousness and injuries) were mentioned by many. Some respondents reported negative emotional effects, such as the loss of emotional control:

“*There was a time when I had an emotional breakdown after a night of drinking. I became so emotional and couldn’t stop crying*”. (21, F, university-educated drinker).

Although negative physical and emotional effects were mentioned as expectancies, negative consequences on interpersonal relationships were not reported, even among heavy drinkers.

#### 3.2.2. Expectancy 2: Confidence Enhancement

The theme of increased confidence was the most frequently stated belief when discussing the expectancies of alcohol. This theme was salient to drinkers of all levels. They believed that drinking made them feel more confident and powerful. The effect was also reported to be beneficial in both social and dating situations, as people found it a lot easier to express themselves and felt confident talking to a member of the opposite sex when they were drunk. As the words of a participant show:

“*I tend to bottle up all my emotions because I don’t want to be shown as weak or emotional. I find that I can only express my grievances in daily life, work, or personnel relations with my friends when I am tipsy*”. (23, M, weekly/binge drinker).

#### 3.2.3. Expectancy 3: Tension Reduction

All participants reported tension reduction or stress release as a benefit of alcohol consumption. They saw drinking as a way to reduce stress for themselves and others. They expected that alcohol consumption could provide a way to relieve tension and stress from school and work. In addition to providing a form of relaxation, some binge drinkers reported using intoxication to escape from their problems. However, they were aware that alcohol can only provide a temporary cure and the problem persisted after they sobered up. In the words of one heavy-drinking participant:

“*The moment I have a drink, I just don’t think [about my problems]. Although the problem persists after I sober up, but that moment of being worry-free helps*”. (22, M, weekly/binge drinker).

#### 3.2.4. Expectancy 4: Social Bonding and Facilitator of Socialization

Social benefits were a positive consequence of engaging in alcohol that was described by all participants. Alcohol was regarded as essential for socializing with peers. Participants felt that they fit into their social circle when they drank with peers and, most importantly, that they were being accepted by others:

“*Drinking helps me fit in in my social group and bond better with my peers. Being able to drink is essential to make friends, especially in [university] residential halls. If I don’t drink, I feel left out*”. (22, F, university-educated drinker).

Moreover, participants found it easier to open up about themselves and make friends when drinking. Therefore, alcohol served as a social lubricant in social settings. Moreover, drinking was considered by some to add to the celebratory atmosphere of social events and improve conviviality:

“*Drinking is a good way to celebrate any special occasion, whether it is birthday or holiday. Having a drink just put you into a better mood*”. (23, F, university-educated drinker).

Drinking was thereby seen as a way to enhance the positive mood of a gathering.

#### 3.2.5. Expectancy 5: Benefits of Business Drinking

Participants believed in a variety of work-related benefits of drinking, particularly among the 25–34-year-old drinkers. In Hong Kong, banquets are common in business settings. Participants found it necessary to drink for corporate events and to socialize with work colleagues:

“*Drinking is necessary for corporate events*”. (27, M, university-educated drinker).

Young non-drinkers also held such an expectancy, as they had seen many business deals come from dinner tables in which alcohol was served as an essential lubricant in movies and television shows. In the words of a participant:

“*Drinking can improve work relationship with colleagues*”. (30, M, non-university-educated drinker).

In addition to serving as a facilitator to work-related functions, many of the respondents also felt that being able to drink would improve their image in the eyes of their work peers and colleagues. Hence, drinking was seen as a “job-skill” by those who wished to be successful in their career (see Table 2).

#### 3.2.6. Expectancy 6: Health Benefits of Drinking

Perceptions of positive health benefits of drinking were prevalent among our group of young adults, particularly among female drinkers. Although the purported cardiovascular benefits of drinking were later clarified to be non-applicable to younger age individuals, other health beliefs were commonly held. Red wine, Japanese plum wine, and traditional Chinese herbal wine were all believed to have health benefits. In addition to the widely promoted health benefits for heart and blood circulation, other health benefit expectancies included skin benefits (especially among female drinkers) and improvements to general body strengthening, similar to views promoted in Traditional Chinese Medicine:

“*I believe that drinking red wine is good for my skin. There are also some Chinese herbal wines that is good for the skin, help detox, and strengthen my body*”. (28, F, non-current drinker).

Additionally, drinking was seen as a way to improve appetite for meals and, thereby, improve health:

“*Some food pairing with drinks is essential. Like you must have Korean fried chicken with beer and steak with wine. Drinking makes me have a greater appetite and eat more*”. (29, F, weekly/binge drinker).

These health benefits appeared to have both specific health benefits (e.g., improved heart health), as well as more general benefits (e.g., improving physical well-being).

**Table 2:** Drinking expectancy domains, definitions, and sample quotes from the Hong Kong young adult focus group participants aged 18–34 (*n* = 53).

Expectancy Domains	Definition	Example of Participant Quotes
Negative consequences	Negative short-term consequences resulted from alcohol consumption (e.g., hangover, poor mood, impaired school/work performance).	***“****Sometimes if I drink too much… there was a period of them when I drink so much that my liver was in pain... and it felt so bad when I have to vomit*”. (20, F, uni educated drinker).
Increased confidence	Alcohol helped to increase self-confidence in social settings and allowed people to be more assertive and less shy.	“*When I went study abroad in Europe, there wasn’t much entertainment, and it was quite bored. So, I went drinking with some of my peers. Surprisingly, I was less concern about getting embarrassed about my English after I had a few drinks down*”. (25, M, non-drinker).
Tension reduction	Alcohol aided in alleviating feelings of stress and tension. Drinking helped people to relax and unwind.	“*When I am usually exhausted after a long day of work. I work at a restaurant and have to stand and serve all day long. I will just grab a drink and relax after work*”. (21, F, non-university-educated drinker).
Social bonding	Alcohol served as a social lubricant to facilitate social interactions and create a sense of closeness in groups. Alcohol eased interpersonal interactions by enlivening the mood of gatherings.	“*I think if I am with a group of friends, alcohol lightens up the atmosphere as it’ll makes you more hyper. Alcohol makes it easier to mingle with others and is always present in my social gatherings*”. (20, F, binge or weekly drinkers).
Business drinking	Work-related benefits to alcohol. Alcohol helped to cement business relationships (with employers, colleagues, and clients) and improved their professional image.	“*I will be seen as more knowledgeable, presentable, and high-class if know my alcohol*”. (21, F, non-drinker).
Health benefits	Belief that alcohol consumption provided certain health benefits (i.e., reduce risk of cardiovascular disease and anti-aging).	“*I have heard some experts say that drinking a glass or a small glass of red wine a day is good for blood circulation and cardiovascular health*”. (26, M, weekly or binge drinker).

### 3.3. Culturally Non-Relevant Expectancies

Although increased sexual interest and cognitive enhancement are common domains included in various survey instruments [10,11], none of the respondents cited these as drinking-related expectancies. When asked about the relevance of these items from a survey instrument, respondents considered these questions to be irrelevant to their own drinking or that of their peer group. Increased sexual interest was not found to be an alcohol expectancy held by any of the focus group participants of our group of Chinese young adults. Most respondents did not report increased sexual interest or engagement in sexual behaviors after drinking. Rather than increasing sexual interest per se, drinking was believed to simply cause an overall increase in impulsive behavior. Similarly, the majority of participants, including non-drinkers, did not see cognitive enhancement as an alcohol expectancy and instead thought that drinking would be more likely to lead to cognitive impairment.

## 4. Discussion

To the best of our knowledge, the present study was the first qualitative study to examine the perceived alcohol expectancies in a Chinese young adult age group. Of the six identified drinking expectancies, four expectancies (i.e., negative consequences, confidence enhancement, tension reduction, and social bonding) had been previously reported in other regions, confirming the importance of these expectancy domains to this age group [10,11,13,58,59]. Two expectancies (i.e., health benefits and business drinking) discussed in our study confirmed past studies, which found unique culturally relevant alcohol expectancies in the Chinese population [60,61,62,63]. However, common expectancies reported in Western cultures (i.e., increased sexual interest and cognitive enhancement) were not seen as relevant in this young adult population.

For this young adult age group, drinking was believed to have interpersonal benefits that extended not only to social settings but also to business/work contexts. Confidence, social bonding, and business drinking were believed to be enhanced by alcohol consumption and were much more important than physical gratification from drinking. This finding is similar to Qian et al.’s study (2018), which found that young Chinese drinkers attached high importance to social/celebratory reasons for drinking [39]. We noted that alcohol was seen as an important social lubricant to help cement relationships that are essential to China’s relationship-based business culture. Many studies reported that the Chinese believe alcohol can help to facilitate business exchange and promote camaraderie between colleagues [60,61,64]. Another study noted that drinkers who were familiar with alcohol, especially wine, were seen as more knowledgeable and presentable among their peers [65]. Hence, among Hong Kong workers, being able to drink is seen as a professional skill that helps one’s career and drinking Western wines/spirits is often seen as part of an aspirational lifestyle. In addition to business drinking, social celebrations, such as weddings, often involve drinking as a social courtesy to the hosts and other guests. Hence, the business and social benefits of drinking that were uncovered in this study indicated that these beliefs are deeply entrenched in Chinese cultural practices, even in young adulthood.

With regard to non-social drinking expectancies, tension reduction and health benefits were the positive expectancies noted in our study, which were counterbalanced by perceptions of the short-term negative physical/emotional effects of drinking. Our findings echoed a recent marketing survey that found that 77% of Chinese consumers cited “health” reasons and 61% of the female respondents cited “beauty” for drinking wine [65]. Although the negative expectancies (e.g., hangover, poor mood) and tension/stress reduction outcomes likely result from personal drinking experience or the observation of drinking peers, the purported effects on health (e.g., reduction in coronary heart disease, lowered mortality rates) are not likely to be personally experienced among adults under 35 years of age. The pervasiveness of these health expectancy beliefs in this age group may simply reflect common traditional Chinese medicine beliefs in Hong Kong that are widely accepted by the populace. In Chinese culture, certain foods/drinks are believed to regulate one vital energy (“qi”) and are consumed as part of daily life to improve health. Alternatively, the health expectancy beliefs may be due to the dissemination of health information in the mass media through popular print magazines and websites. A past study noted that many people in Hong Kong obtain health information from popular mass media rather than government sources or recognized scientific sources [66]. The absence of reliable health information sources to counterbalance the purported benefits may have contributed to the misconception about alcohol-related health benefits.

In contrast to findings from Western studies [67,68,69], drinking was not seen as a way to decrease sexual inhibitions or enhance a sexual experience. Both the AEQ and DEQ have domains related to sexual enhancement, but this domain was not deemed to be an expectancy by our respondents. Our findings echoed the results of cross-ethnic and cross-cultural studies of Caucasians and Asian young adults that reported lower sexual expectancies and less frequent alcohol-involved sex among Asian samples [34,70]. While the use of psychoactive substances was often associated with cognitive enhancement in Western countries [69], one possible reason why these expectancies were not reported in our study may partially be due to the high prevalence of flushing reaction in Southern Chinese [71]. Alcohol consumption is not an integral part of dating customs in Hong Kong and alcohol flushing is generally considered undesirable to one’s appearance, which may reduce its use in romantic contexts [72]. Moreover, heavy drinking is typically done in Hong Kong in large groups of friends or co-workers, which may also reduce the associations with sexual encounters. The lower alcohol tolerance among many drinkers also may reduce any cognitive benefits. Moreover, binge drinking among such drinkers may simply result in cognitive impairment rather than cognitive enhancement.

Previous research found that positive drinking expectancies mediate the effect of alcohol advertising on drinking behaviors [17,18,19,20]. Alcohol marketing in Hong Kong, particularly digital marketing, commonly associates drinking with celebratory events, friendship, life achievement, and an aspirational lifestyle, which correlated with many of the expectancies uncovered in our study, such as social bonding, tension reduction, increased confidence, and work-related drinking. Public health interventions directed at this age group, therefore, need to address the positive expectancies promoted by alcohol marketing and emphasize the negative consequences of drinking. This age group is legally allowed to purchase and consume alcohol, and thus, the measures that will likely reduce alcohol-related harms based on the current evidence are increasing the price of alcohol (via taxation or minimum pricing laws) and regulation of alcohol marketing [73,74]. Alcohol marketing currently has extremely limited regulation in traditional mass media and virtually no regulation on social media [75]. Policymakers may consider implementing content-based marketing regulations to restrict content that further reinforces the linkage between drinking and positive alcohol expectancies (e.g., tension reduction, social bonding, and business drinking). Public health educators can also leverage social media platforms to produce broad-reaching health messages to counterbalance persuasive advertisement messages and reduce misconceptions about the positive benefits of alcohol.

This study had several limitations. First, the small number of participants might not fully represent the views of Hong Kong young adults. However, care was taken to include a range of drinking levels, educational backgrounds, and occupations. Nonetheless, our focus group participants had somewhat lower levels of disposable income (based on their household incomes) than those in the general population of young adults. The drinking expectancies of high-income young adults may differ and may need to be examined in future studies. Second, the young adults’ drinking expectancies may not be generalizable to other age groups. However, the purpose of this study was to examine the association of drinking behaviors in an age group that is heavily targeted by alcohol marketing and most at risk of alcohol harm. Third, the motivations of the non-drinkers were not examined. Future studies may, therefore, examine the factors that influence the non-uptake of drinking, as well as the cessation of drinking behaviors in young adults. Fourth, because the study was based on self-reported data, the findings might be influenced by social desirability biases. Nonetheless, with confidentiality and anonymity assurances, these biases should be moderate. Lastly, although China has experienced a period of rapid development, during which young and affluent drinkers are part of an emerging drinking culture, the experiences of Hong Kong drinkers may not fully reflect those in other cities in China. Studies conducted on Chinese adolescents found that the adoption of Western values and cultural orientation increased positive drinking expectancies [76,77]. Since the lifestyle habits of Hong Kong often foreshadow those in other parts of China, the results of this study may bring insights for research in other parts of China.

This research was an initial exploratory study to examine commonly held alcohol expectancies among Chinese young adults. Further analysis will be performed on the result to generate a culturally relevant alcohol expectancy questionnaire. Future applications of the questionnaire in health promotion, clinical settings, and research may contribute to reducing the harm associated with alcohol use among young adults. The results of this initial study may not be immediately generalizable to the larger population, but it may serve as a springboard for future longitudinal studies and initiate dialogue to continue to examine culturally unique factors that influence young people’s drinking. Future longitudinal studies should also examine the changes in young adults’ alcohol expectancies before and after their exposure to alcohol marketing to establish the influence of alcohol advertisements on viewers’ drinking expectancies. The study also highlighted the fact that alcohol expectancies were highly influenced by social and cultural norms. Hence, it is recommended that culture-specific drinking expectancy tools be developed and used for assessing drinking behaviors in non-Western countries.

## 5. Conclusions

In light of the present findings, prevention programs that target young adults’ drinking should address the commonly held expectancies of alcohol use. The combination of self-perceived health and interpersonal benefits of drinking under the backdrop of limited alcohol marketing regulation reinforced the positive perceptions about alcohol consumption. The culturally relevant expectancies uncovered in this study should be considered in future policy discussions about alcohol marketing regulations in this region.

## Figures and Tables

**Table 1:** Demographic characteristics of the Hong Kong young adult focus group participants aged 18–34 (*n* = 53).

Demographic Characteristics	% (*n*)
**Gender**	
Male	49.1% (26)
Female	50.9% (27)
**Age**	
18–24	52.8% (28)
25–29	37.7% (20)
30–34	9.4% (5)
**Educational attainment**	
Up to F.5	1.9% (1)
Higher diploma/associate degree	32.1% (17)
University or higher	66.0% (35)
**Experienced living abroad**	
No	62.3% (33)
Yes	37.7% (20)
**Marital status**	
Single	90.6% (48)
Cohabitating	3.8% (2)
Married	5.7% (3)
**Employment status**	
Unemployed/housewife/househusband	15.1% (8)
Full-time student	41.5% (22)
Employed	43.4% (23)
**Monthly household income** (HKD 7.8 = USD 1)	
Under HKD 20,000	26.4% (14)
HKD 20,000–39,999	39.6% (21)
HKD 40,000–59,999	15.1% (8)
HKD 60,000 or above	18.9% (10)

## Data Availability

The video-recorded data presented in this study are available on request from the corresponding author. The data are not publicly available in order to maintain the privacy of focus group participants.

## References

[1] World Health Organization (2018). Global Status Report on Alcohol and Health 2018.

[2] Jiang H., Xiang X., Hao W., Room R., Zhang X., Wang X. (2018). Measuring and preventing alcohol use and related harm among young people in Asian countries: A thematic review. Glob. Health Res. Policy.

[3] Boyd G.M., Howard J., Zucker R.A. (1995). Alcohol Problems among Adolescents: Current Directions in Prevention Research.

[4] Goldman M., Del Boca F., Darkes J. (1999). Alcohol Expectancy Theory: The Application of Cognitive Neuroscience. Psychological Theories of Drinking and Alcoholism.

[5] Zamboanga B.L., Schwartz S.J., Ham L.S., Jarvis L.H., Olthuis J.V. (2009). Do alcohol expectancy outcomes and valuations mediate peer influences and lifetime alcohol use among early adolescents?. J. Genet. Psychol..

[6] Ting T.T., Chen W.J., Liu C.Y., Lin Y.C., Chen C.Y. (2015). Peer influences on alcohol expectancies in early adolescence: A study of concurrent and prospective predictors in Taiwan. Addict. Behav..

[7] Smit K., Voogt C., Hiemstra M., Kleinjan M., Otten R., Kuntsche E. (2018). Development of alcohol expectancies and early alcohol use in children and adolescents: A systematic review. Clin. Psychol. Rev..

[8] Wills T.A., Sargent J.D., Gibbons F.X., Gerrard M., Stoolmiller M. (2009). Movie Exposure to Alcohol Cues and Adolescent Alcohol Problems: A Longitudinal Analysis in a National Sample. Psychol. Addict. Behav..

[9] Christiansen B.A., Smith G.T., Roehiing P.V., Goldman M.S. (1989). Using Alcohol Expectancies to Predict Adolescent Drinking Behavior After One Year. J. Consult. Clin. Psychol..

[10] Brown S.A., Christiansen B.A., Goldman M.S. (1987). The Alcohol Expectancy Questionnaire: An instrument for the assessment of adolescent and adult alcohol expectancies. J. Stud. Alcohol.

[11] Lee N.K., Oei T.P.S., Greeley J.D., Baglioni A.J. (2003). Psychometric properties of the drinking expectancy questionnaire: A review of the factor structure and a proposed new scoring method. J. Stud. Alcohol.

[12] Young R.M.D., Knight R.G. (1989). The Drinking Expectancy Questionnaire: A revised measure of alcohol-related beliefs. J. Psychopathol. Behav. Assess..

[13] Young R.M.D., Connor J.P., Ricciardelli L.A., Saunders J.B. (2006). The role of alcohol expectancy and drinking refusal self-efficacy beliefs in university student drinking. Alcohol Alcohol..

[14] Vilenne A., Quertemont E. (2015). Explicit and Implicit Positive Alcohol Expectancies in Problem and Non-Problem Drinkers: Differences Across Age Groups from Young Adolescence to Adulthood. Front Psychol..

[15] Ham L.S., Hope D.A. (2003). College students and problematic drinking: A review of the literature. Clin. Psychol. Rev..

[16] Cameron C.A., Stritzke W.G.K., Durkin K. (2003). Alcohol expectancies in late childhood: An ambivalence perspective on transitions toward alcohol use. J. Child Psychol. Psychiatry Allied Discip..

[17] de Graaf A. (2013). Alcohol Makes Others Dislike You: Reducing the Positivity of Teens’ Beliefs and Attitudes Toward Alcohol Use. Health Commun..

[18] Elmore K.C., Scull T.M., Kupersmidt J.B. (2017). Media as a “Super Peer”: How Adolescents Interpret Media Messages Predicts Their Perception of Alcohol and Tobacco Use Norms. J. Youth Adolesc..

[19] Ho S.S., Poorisat T., Neo R.L., Detenber B.H. (2014). Examining how presumed media influence affects social norms and adolescents’ attitudes and drinking behavior intentions in rural Thailand. J. Health Commun..

[20] Morgenstern M., Schoeppe F., Campbell J., Braam M.W.G., Stoolmiller M., Sargent J.D. (2015). Content Themes of Alcohol Advertising in U.S. Television-Latent Class Analysis. Alcohol Clin. Exp. Res..

[21] Chen C.Y., Huang H.Y., Tseng F.Y., Chiu Y.C., Chen W.J. (2017). Media alcohol advertising with drinking behaviors among young adolescents in Taiwan. Drug Alcohol Depend..

[22] Shell D.F., Newman I.M., Qu M. (2009). Alcohol expectancies among high school students in Inner Mongolia, China. Asia-Pac. J. Public Health.

[23] Newman I., Shell D.F., Newman I.M. (2005). Alcohol Expectancies among a Sample of Thai High School Students of Thai High School Students. Educ. Psychol..

[24] Mou Y., Lin C.A. (2020). Consuming Alcohol to Prepare for Adulthood: An Event History Analysis of the Onset of Alcohol Use Among Chinese College Students. SAGE Open.

[25] Yoo H.H., Cha S.W., Lee S.Y. (2020). Patterns of alcohol consumption and drinking motives among korean medical students. Med. Sci. Monit..

[26] Nadkarni A., Tu A., Garg A., Gupta D., Gupta S., Bhatia U., Tiwari N., Heath A., Wen K., Fernandes G. (2022). Alcohol use among adolescents in India: A systematic review. Glob Ment Health.

[27] Arnett J.J. (2000). Emerging adulthood: A theory of development from the late teens through the twenties. Am. Psychol..

[28] Petry N.M. (2002). A comparison of young, middle-aged, and older adult treatment-seeking pathological gamblers. Gerontologist.

[29] ProCon.org (2016). Minimum Legal Drinking Age in Other Countries. https://drinkingage.procon.org/minimum-legal-drinking-age-in-other-countries/.

[30] Tang Y., Xiang X., Wang X., Cubells J.F., Babor T.F., Hao W. (2013). Alcohol and alcohol-related harm in China: Policy changes needed. Bull. World Health Organ..

[31] Manthey J., Shield K.D., Rylett M., Hasan O.S.M., Probst C., Rehm J. (2019). Global alcohol exposure between 1990 and 2017 and forecasts until 2030: A modelling study. Lancet..

[32] Lu W., Xu J., Taylor A.W., Bewick B.M., Fu Z., Wu N., Qian L., Yin P. (2019). Analysis of the alcohol drinking behavior and influencing factors among emerging adults and young adults: A cross-sectional study in Wuhan, China. BMC Public Health..

[33] Ahn S. (2012). Exploring Alcohol Expectancies in Korea and America Using the Holism Theory. Master’s Thesis.

[34] Oei T.P.S., Jardim C.L. (2007). Alcohol expectancies, drinking refusal self-efficacy and drinking behaviour in Asian and Australian students. Drug Alcohol Depend..

[35] O’Hare T. (1995). Differences in asian and white drinking: Consumption level, drinking contexts, and expectancies. Addict. Behav..

[36] Kim H.K., En R.L.S., Min D.W.K. (2019). Psychosocial motivators for moderate drinking among young Asian flushers in Singapore. Int. J. Environ. Res. Public Health.

[37] Zhang M.X., Ku L., Wu A.M.S., Yu S.M., Pesigan I.J.A. (2020). Effects of Social and Outcome Expectancies on Hazardous Drinking among Chinese University Students: The Mediating Role of Drinking Motivations. Subst. Use Misuse..

[38] Taylor A.W., Bewick B.M., Ling Q., Kirzhanova V., Alterwain P., Dal Grande E., Tucker G., Makanjuola A.B. (2019). Assessing heavy episodic drinking: A random survey of 18 to 34-year-olds in four cities in four different continents. Int. J. Environ. Res. Public Health.

[39] Qian L., Newman I.M., Yuen L.W., Shell D.F., Xu J. (2018). Variables associated with alcohol consumption and abstinence among young adults in central China. Int. J. Environ. Res. Public Health.

[40] Census and Statistics Department HKSAR (2021). Hong Kong Annual Digest of Statistics 2020 Edition. https://www.scopus.com/inward/record.uri?eid=2-s2.0-84898235993&partnerID=40&md5=79d92483774b256de146cb564c089b4d.

[41] Hong Kong Department of Health (2018). Alcohol Consumption per Capita in HK 2004-2017_Eng.

[42] Hong Kong Department of Health (2011). Alcohol and Health: Hong Kong Situation. https://www.google.com.hk/url?sa=t&rct=j&q=&esrc=s&source=web&cd=&cad=rja&uact=8&ved=2ahUKEwiqlcrM-6T6AhXrmFYBHf6BBkgQFnoECAgQAQ&url=https%3A%2F%2Fwww.dh.gov.hk%2Fenglish%2Fpub_rec%2Fpub_rec_ar%2Fpdf%2Fncd_ap2%2Faction_plan_2_alcohol%2520and%2520health%2520HK%2520situation_e.pdf&usg=AOvVaw219AF1f2B8i106NBTF9trI.

[43] Hong Kong Department of Health (2015). Report of Population Health Survey 2014/15.

[44] Chung V.C.H., Yip B.H.K., Griffiths S.M., Yu E.L.M., Kim J.H., Tam W.W.S., Wong A.H.C., Chan I.W.T., Lau J.T.F. (2013). The impact of cutting alcohol duties on drinking patterns in Hong Kong. Alcohol Alcohol..

[45] Kim J.H., Chan K.W.C., Chow J.K.W., Fung K.P., Fong B.Y.F., Cheuk K.K., Griffiths S.M. (2009). University binge drinking patterns and changes in patterns of alcohol consumption among chinese undergraduates in a Hong Kong university. J. Am. Coll. Health.

[46] Kim J.H., Lee S., Chow J., Lau J., Tsang A., Choi J., Griffiths S.M. (2008). Prevalence and the factors associated with binge drinking, alcohol abuse, and alcohol dependence: A population-based study of chinese adults in Hong Kong. Alcohol Alcohol..

[47] Yu J., Sumerlin T.S., Goggins W.B., Dong D., Chung R.Y.N., Kim J.H. (2022). First- and second-hand alcohol-related harms among urban Chinese: A population-based study from Hong Kong. Drug Alcohol Rev..

[48] Arain M., Haque M., Johal L., Mathur P., Nel W., Rais A., Sandhu R., Sharma S. (2013). Maturation of the adolescent brain. Neuropsychiatr. Dis. Treat..

[49] Lau J.T., Kim J.H., Tsui H.Y. (2005). Prevalence, health outcomes, and patterns of psychotropic substance use in a Chinese population in Hong Kong: A population-based study. Subst. Use Misuse..

[50] Kim J.H., Wong A.H., Goggins W.B., Lau J., Griffiths S.M. (2013). Drink driving in Hong Kong: The competing effects of random breath testing and alcohol tax reductions. Addiction.

[51] Kim J.H., Lee S., Chan K.W., Lau J., Tsang A., Griffiths S.M. (2010). A population-based study on the prevalence and correlates of drinking and driving in Hong Kong. Accid. Anal. Prev..

[52] Creswell J., Guetterman T. (2018). Educational Research: Planning, Conducting, and Evaluating Quantitative and Qualitative Research.

[53] Collins S.E. (2016). Associations between socioeconomic factors and alcohol outcomes. Alcohol Res. Curr. Rev..

[54] Murakami K., Hashimoto H. (2019). Associations of education and income with heavy drinking and problem drinking among men:Evidence from a population-based study in Japan. BMC Public Health..

[55] Barbu C.G. (2014). Zoom: A spatial data visualization tool. https://cran.r-project.org/web/packages/zoom/zoom.pdf.

[56] Kahan J.P. (2001). Focus Groups as a Tool for Policy Analysis. Anal. Soc. Issues Public Policy.

[57] (2011). NVivo Qualitative Data Analysis Software.

[58] Pabst A., Baumeister S.E., Kraus L. (2010). Alcohol-expectancy dimensions and alcohol consumption at different ages in the general population. J. Stud. Alcohol Drugs.

[59] Leeman R.F., Toll B.A., Taylor L.A., Volpicelli J.R. (2009). Alcohol-induced disinhibition expectancies and impaired control as prospective predictors of problem drinking in undergraduates. Psychol. Addict. Behav..

[60] Cochrane J., Chen H., Conigrave K.M., Hao W. (2003). Alcohol use in China. Alcohol Alcohol..

[61] Hao W., Chen H., Su Z. (2005). China: Alcohol today. Addiction.

[62] Qian L., Newman I.M., Xiong W., Feng Y. (2015). Traditional grain alcohol (bai jiu), production and use in rural central China: Implications for public health. BMC Public Health..

[63] Metcalf D.A., Saliba A., McKenzie K., Gao A. (2021). Relationships between consumption patterns, health beliefs, and subjective wellbeing in Chinese Baijiu consumers. Subst. Abus. Treat. Prev. Policy.

[64] Hao W., Young D. (2000). Drinking patterns and problems in China. J. Subst. Use.

[65] Poon C. (2020). China’s Wine Market Consumer Preferences (1): Wine Category, Drinking Occasion and Price. HKTDC Research. https://research.hktdc.com/en/article/NTgzNTI4NDg3.

[66] Kim J.H., Kwong E.M.S., Chung V.C.H., Lee J.C.O., Wong T., Goggins W.B. (2013). Acute adverse events from over-the-counter Chinese herbal medicines: A population-based survey of Hong Kong Chinese. BMC Complement Altern Med..

[67] McKay A. (2005). Sexuality and substance use: The impact of tobacco, alcohol, and selected recreational drugs on sexual function. Can. J. Hum. Sex.

[68] Patrick M.E., Maggs J.L., Lefkowitz E.S. (2015). Daily Associations between Drinking and Sex among College Students: A Longitudinal Measurement Burst Design. J. Res. Adolesc..

[69] Maier L.J., Ferris J.A., Winstock A.R. (2018). Pharmacological cognitive enhancement among non-ADHD individuals—A cross-sectional study in 15 countries. Int. J. Drug Policy.

[70] Dir A.L., Andrews A.R., Wilson S.M., Davidson T.M., Gilmore A.K. (2018). The Role of Sex-Related Alcohol Expectancies in Alcohol-Involved Consensual and Nonconsensual Sex among Women of Asian/Pacific Islander and Women of European Race/Ethnicity. J Sex Res..

[71] Eng M.Y., Luczak S.E., Wall T.L. (2007). ALDH2, ADH1B, and ADH1C genotypes in Asians: A literature review. Alcohol Res. Health.

[72] Wall T.L., Ehlers C.L. (1995). Genetic Influences Affecting Alcohol Use Among Asians. Alcohol Health Res. World.

[73] Elder R.W., Lawrence B., Ferguson A., Naimi T.S., Brewer R.D., Chattopadhyay S.K., Toomey T.L., Fielding J.E. (2010). The Effectiveness of Tax Policy Interventions for Reducing Excessive Alcohol Consumption and Related Harms. Am. J. Prev. Med..

[74] Esser M.B., Jernigan D.H. (2018). Policy Approaches for Regulating Alcohol Marketing in a Global Context: A Public Health Perspective. Annu. Rev. Public Health.

[75] Room R., O’Brien P. (2021). Alcohol marketing and social media: A challenge for public health control. Drug Alcohol Rev..

[76] Shell D.F., Newman I.M., Fang X. (2010). The Influence of Cultural Orientation, Alcohol Expectancies, and Self-Efficacy on Adolescent Drinking Behavior in Beijing. Addiction.

[77] Qian L., Hu T., Newman I.M., Hou P.S. (2008). Study on the relationships between cultural orientation, alcohol expectancy, self-efficacy and drinking behavior among senior high school students in two cities of Henan province. Zhonghua Liu Xing Bing Xue Za Zhi.

